# Pre- and Postovulatory Aging of Murine Oocytes Affect the Transcript Level and Poly(A) Tail Length of Maternal Effect Genes

**DOI:** 10.1371/journal.pone.0108907

**Published:** 2014-10-01

**Authors:** Debora Dankert, Hannah Demond, Tom Trapphoff, Martyna Heiligentag, Katrin Rademacher, Ursula Eichenlaub-Ritter, Bernhard Horsthemke, Ruth Grümmer

**Affiliations:** 1 Institute of Anatomy, University Hospital Essen, University Duisburg-Essen, Essen, Germany; 2 Institute of Human Genetics, University Hospital Essen, University Duisburg-Essen, Essen, Germany; 3 Institute of Gene Technology/Microbiology, University of Bielefeld, Bielefeld, Germany; China Agricultural University, China

## Abstract

Maternal effect genes code for oocyte proteins that are important for early embryogenesis. Transcription in oocytes does not take place from the onset of meiotic progression until zygotic genome activation. During this period, protein levels are regulated posttranscriptionally, for example by poly(A) tail length. Posttranscriptional regulation may be impaired in preovulatory and postovulatory aged oocytes, caused by delayed ovulation or delayed fertilization, respectively, and may lead to developmental defects. We investigated transcript levels and poly(A) tail length of ten maternal effect genes in in vivo- and in vitro- (follicle culture) grown oocytes after pre- and postovulatory aging. Quantitative RT-PCR was performed using random hexamer-primed cDNA to determine total transcript levels and oligo(dT)_16_-primed cDNA to analyze poly(A) tail length. Transcript levels of in vivo preovulatory-aged oocytes remained stable except for decreases in *Brg1* and *Tet3*. Most genes investigated showed a tendency towards increased poly(A) content. Polyadenylation of in vitro preovulatory-aged oocytes was also increased, along with transcript level declines of *Trim28*, *Nlrp2*, *Nlrp14* and *Zar1*. In contrast to preovulatory aging, postovulatory aging of in vivo- and in vitro-grown oocytes led to a shortening of poly(A) tails. Postovulatory aging of in vivo-grown oocytes resulted in deadenylation of *Nlrp5* after 12 h, and deadenylation of 4 further genes (*Tet3, Trim28, Dnmt1*, *Oct4*) after 24 h. Similarly, transcripts of in vitro-grown oocytes were deadenylated after 12 h of postovulatory aging (*Tet3, Trim28, Zfp57, Dnmt1, Nlrp5*, *Zar1*). This impact of aging on poly(A) tail length may affect the timed translation of maternal effect gene transcripts and thereby contribute to developmental defects.

## Introduction

The growing vertebrate oocyte accumulates maternal transcripts and proteins required for oocyte maturation, fertilization, zygotic genome activation (ZGA) and early embryogenesis. Since transcription of the maternal genome is shut down at the onset of meiotic progression, synthesis of new proteins before ZGA is regulated exclusively at the posttranscriptional level, for example by alterations in the poly(A) tail length of mRNAs that are involved in recruitment and degradation of mRNAs [1], [2], [3]. The length of the poly(A) tail is a major factor in eukaryotic posttranscriptional regulation, since polyadenylation protects mRNAs from degradation and is required for efficient translation [4], [5]. Maternal effect (ME) genes encode proteins that are determined by the maternal genotype and affect the phenotype of the offspring. They are important for the regulation of the transition of highly specialized gametes to totipotent zygotes [6], which involves recruitment and degradation of maternal mRNA and proteins, DNA demethylation and histone modifications, while protecting and maintaining genomic imprinting [7], [8], [9], [10], [11]. So far, over 30 ME genes have been described in mice [12].

The developmental competence of the oocyte and the early embryo is highly sensitive and may be seriously disturbed by changes in the physiological time axis prior to as well as after ovulation. Pre- or postovulatory aging of the oocyte is suspected to affect oocyte quality and can occur at any age in fertile females as well as during assisted reproductive techniques. Preovulatory aging results from delayed ovulation and is known to cause developmental defects, such as loss of implantation potential, malformations and high mortality rates in various animal species [13] as well as an increase in embryo resorption sites and decline in embryonic weight in mice [14]. The molecular mechanisms underlying these developmental defects are so far unknown.

Postovulatory aging is caused by delayed fertilization. Oocytes arrested at the metaphase II (MII) stage are normally fertilized soon after ovulation. If fertilization does not occur within that time; unfertilized oocytes will undergo “postovulatory oocyte aging”, a time-dependent deterioration in quality. It has been shown in numerous studies that postovulatory aging leads to abnormal development and dramatically reduced oocyte quality in frogs as well as various laboratory mammals [15], [16], [17], [18], [19], [20]. One major cause for impaired developmental competence after postovulatory aging of oocytes is oxidative stress, which triggers many cascades that influence oocyte quality, like mitochondrial dysfunction, DNA damage and perturbed Ca^2+^ homeostasis [21], [22], [23], [24]. Sister chromatid cohesion and the microtubular cytoskeleton and spindle may also be affected by prolonged culture and in vitro aging, for instance during postovulatory aging of human MII oocytes [25]. We postulate that aberrant posttranscriptional regulation of mRNA, such as deadenylation or degradation of maternal transcripts during postovulatory aging, is another mechanism that may reduce oocyte quality. Adenylation dynamics of mRNA are known to be strictly regulated [3], [26], although only few studies have addressed the dynamics of these processes in the mammalian oocyte with regard to ME genes [27]. It has been shown that some mRNAs in cell cycle regulation appear to possess a longer poly(A) tail in in vitro-matured, cumulus-depleted human oocytes with restricted developmental potential compared to in vivo-matured MII oocytes [3], [26]. It has recently been shown for *Xenopus tropicalis* that postovulatory aging of the oocyte leads to deadenylation of a variety of ME genes and serious malformations of the larvae [28]. Whether this regulation also plays a role in mammals is not known yet. Moreover, suboptimal culture conditions, in particular changes in duration of growth and maturation of oocytes in a follicle culture system, have previously been shown to reduce oocyte quality, leading to low fertilization rates and compromising the developmental potential of the mouse oocyte [29], [30].

In the current study, we investigated the effects of pre- and postovulatory aging on total transcript levels and adenylation levels as indicated by poly(A) tail length of ten different ME genes in a mouse model in vivo as well as after in vitro growth and maturation of mouse oocytes in a preantral follicle culture model [31].

## Materials and Methods

### Ethics Statement

This study was carried out in strict accordance with the recommendations in the Guide for the Care and Use of Laboratory Animals of the German Government. The protocol was approved by the Committee on the Ethics of Animal Experiments of the responsible authorities (Landesamt für Natur, Umwelt und Verbraucherschutz, LANUV AZ 84-02.04.2011.A374).

### Generation of pre- and postovulatory-aged mouse oocytes grown in vivo

**Figure 1:**
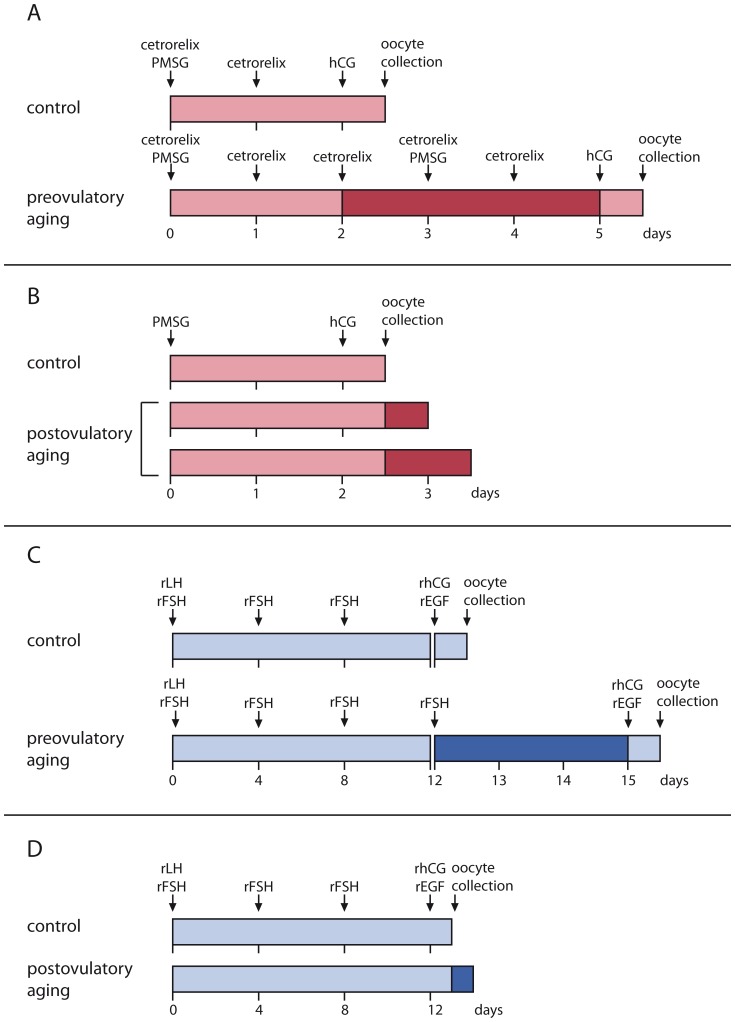
Timeline of pre- and postovulatory aging in vivo (A, B) and in vitro (C, D). A, B) For in vivo maturation of oocytes, follicle maturation was stimulated by PMSG on day 0. Ovulation was induced by hCG 48 h later. Control oocytes were collected from the ampullae the next morning. For preovulatory in vivo aging, ovulation was delayed by the GnRH antagonist cetrorelix for 3 d (A). Oocytes for postovulatory aging were collected at the same time as controls and cultured in M2 medium for further 12 or 24 h (B). C, D) For in vitro growth and maturation of oocytes, preantral follicles were cultured for 12 d in the presence of rLH and rFSH to the antral follicle stage. Ovulation was induced with rhCG/rEGF and control oocytes were collected after 18 h. To obtain preovulatory-aged oocytes, ovulation was induced with rhCG/rEGF on day 15 of follicle culture instead of day 12 (C). For postovulatory aging, ovulation was triggered with rhCG/rEGF and oocytes were incubated for additional 12 h before collection (D).

For in vivo maturation of oocytes, 4–6 week old C57Bl/6J female mice were maintained under standard conditions (12 h light and dark cycles, food and water ad libitum). Preovulatory aging of oocytes can be achieved by prolonged duration of oocyte maturation prior to ovulation, leading to oocyte overripeness. To obtain preovulatory-aged oocytes, ovulation in mice was delayed by application of the GnRH antagonist Cetrorelix (Cetrotide, Merck-Serono), as previously described [14]. In short, during stimulation of follicle growth by i.p. injection of 10 IU pregnant mare serum gonadotropin (PMSG; Intervet), in parallel Cetrorelix (50 µg/day) was applied s.c. to prevent endogenous triggering of spontaneous ovulation. To obtain oocytes with regular duration of preovulatory maturation (controls), ovulation was induced by i.p. injection of 10 IU human chorionic gonadotropin (hCG; Intervet) 48 h after PMSG and Cetrorelix application. Mice were sacrificed by cervical dislocation 15–17 h after hCG injection. Oviducts were removed and cumulus-oocyte-complexes (COCs) were collected from the oviductal ampullae. All cumulus cells were removed from the oocytes enzymatically by hyaluronidase treatment (Sigma-Aldrich) for 2 min and oocytes were subsequently washed in M2 medium (Sigma-Aldrich). Oocytes were immediately frozen at −80°C. To obtain preovulatory-aged oocytes, Cetrorelix treatment was prolonged to 5 days and follicle stimulation was maintained by a second injection of PMSG 48 h after the beginning of treatment (Fig. 1A). Ovulation was induced by i.p. injection of 10 IU hCG on the evening of day 5 of treatment resulting in a 3-day delay in ovulation compared to controls.

Postovulatory aging of oocytes is defined as a prolonged time frame between ovulation and fertilization. To obtain oocytes for postovulatory aging, oocyte maturation was stimulated in mice by i.p. injection of 10 IU PMSG. Ovulation was induced by i.p. injection of 10 IU hCG 48 h later. COCs were collected 15–17 h after hCG-injection from the oviductal ampullae and all cumulus cells were removed as described above. Metaphase II oocytes from each mouse were split into two equivalent groups. Oocytes of one group were immediately frozen at −80°C (controls). Oocytes of the other group were transferred to CO_2_-equilibrated M2 medium covered with mineral oil (Sigma-Aldrich) and were aged for 12 or 24 h at 37°C and 5% CO_2_ before being stored at −80°C (Fig. 1B).

### Generation of pre- and postovulatory-aged mouse oocytes grown in vitro

For in vitro growth (IVG) and in vitro maturation (IVM) of oocytes, preantral follicles were mechanically isolated from the ovaries of 12- to 14-day-old female F1 hybrid C57Bl/6J × CBA/Ca mice. Follicle culture was performed as previously described [31], [32], [33]. In brief, preantral follicles (3b follicles; [34]) were cultured at 37°C and 5% CO_2_ in α-MEM Glutamax (Invitrogen) supplemented with 10 mIU/ml recombinant follicle-stimulating hormone (rFSH; Merck-Serono), 5% fetal calf serum, 5 µg/ml insulin, 5 µg/ml transferrin, 5 ng/ml sodium selenite (Sigma-Aldrich) and covered with mineral oil (Invitrogen). Recombinant luteinizing hormone (rLH; 10 mIU/ml kindly donated by Merck-Serono) was added at the beginning of the in vitro follicle culture [31]. On every fourth day, the 20 µl of medium with rFSH were replenished.

For IVG oocyte controls, in vitro maturation and ovulation was induced on day 12 of culture by 5 ng/ml recombinant epidermal growth factor (rEGF, Promega) and 1.5 IU/ml rhCG (kindly donated by Merck-Serono). COCs containing MII oocytes were harvested on day 13 (18 h post rhCG/rEGF, control).

To generate preovulatory-aged oocytes, the culture medium was replenished on day 12 and resumption of maturation and ovulation was induced with a delay on day 15 instead of day 12 of culture. COCs were harvested on the following day (18 h post rhCG/rEGF; Fig. 1C). Postovulatory aging was induced by harvest on day 13 plus 12 h of aging (30 h after initiation of rEGF/rhCG treatment; Fig. 1D). For all IVM groups, cumulus cells were removed immediately before oocyte retrieval by brief hyaluronidase treatment, and MII oocytes were stored at −80°C as described for in vivo samples.

Follicle developmental kinetics were assessed at day 4, 8, 12 and 15 of follicle culture and classified into three groups as previously described [35]. In brief, stage one (“follicular”) contains the centrally located oocyte with two to three granulosa cell layers and some outgrowing theca- and granulosa cells. In stage two (“diffused”) the spherical follicle conformation is lost and granulosa cells proliferate and flatten out on the culture well. In stage three (“antrum”) cumulus and mural granulosa cell differentiation and formation of antral-like-cavity structures becomes visible. The maturation rate was determined by the number of oocytes with a germinal vesicle (GV), germinal vesicle breakdown (GVBD) and first polar body (MII) after rEGF/rhCG stimulation at time of harvest.

Concentration of estrogen and progesterone was analyzed in replenished medium from different culture conditions using competitive immunoassays (Centaur XP, Siemens). Control group samples were obtained prior to rhCG/rEGF treatment on day 12 and post-treatment on day 13. Preovulatory group samples were obtained before and after rhCG/rEGF treatment on days 13 and 16, respectively. Postovulatory aged samples were collected on day 13 post in vitro ovulation and after 12 h of aging.

### RNA Extraction

Total RNA was prepared from pools of 20 oocytes each using the Arcturus PicoPure RNA Isolation Kit (Life Technologies) according to manufacturer's instructions with minor changes: Prior to RNA isolation, 1 pg Luciferase RNA/oocyte (Promega) was added to each sample as a reference for RNA quantification during qRT-PCR analysis [36], since housekeeping genes are not reliable as reference as it is unknown whether they are affected by oocyte aging. Oocytes were incubated in the provided extraction buffer for 30 min at 42°C and were subsequently homogenized using a QiaShredder Column (Qiagen). During RNA isolation, samples were depleted from genomic DNA with treatment of DNase I (Qiagen) according to the protocol.

### qRT-PCR analysis

Overall transcript levels and poly(A) content of ten ME genes were determined by qRT-PCR. Total RNA from three pools of 20 oocytes from each experimental group was reverse transcribed using MuLV reverse transcriptase (Life Technologies). Overall transcript levels were determined using random hexamer-primed (Life Technologies) cDNA. Oligo(dT)_16_-primed (Life Technologies) cDNA was used as an indicator for poly(A) tail length. RNA from different oocyte pools was used for random hexamer and oligo(dT)_16_ priming.

Quantitative RT-PCR was performed with the Light Cycler 480 II (Roche) using either the Universal Probe Liberary (UPL; Roche) or Taqman gene expression assays (Life Technologies) to analyze expression of *Brg1* (*Smarca4*), *Tet3*, *Trim28* (*Kap1*/*Tif1β*), *Zfp57*, *Dnmt1*, *Nlrp2*, *Nlrp5* (*Mater*), *Nlrp14*, *Oct4* (*Pou5f1*) and *Zar1*. Luciferase was used as an external reference. Primers provided by Biomers, Taqman assays and UPL probes are listed in Table 1. After incubation for 10 min at 95°C, transcripts were amplified using 45 cycles of 10 s at 95°C, 30 s at 60°C and 1 s at 72°C. Each sample was measured in triplicate. The normalized fold change of aged oocytes compared to controls was determined using the 2^-ΔΔCt^ method.

**Table 1:** qRT-PCR primer sequences.

Gene	Ref-Seq transcript ID	Primer sequence (5′–3′)		UPL-Probe
*Brg1*	NM_011417.3	F	cggttgtgagtgacgatgac	15
		R	cctcactgccacttcctga	
*Tet3*	NM_183138.2	F	gcacgccagagaagatcaa	81
		R	ggacaatccacccttcagag	
*Trim28*	NM_011588.3		Taqman assay (Mm00495594_m1)	
*Zfp57*	NM_001013745.2	F	aaccttcaagaacctgacatttg	15
		R	ccctgtgcaactggagga	
*Dnmt1*	NM_001199431.1		Taqman assay (Mm01151063)	
*Nlrp2*	NM_117690.3		Taqman assay (Mm00624616_m1)	
*Nlrp5*	NM_001039143.1		Taqman assay (Mm011143609_m1)	
*Nlrp14*	NM_001002894.2	F	gcagccacactgcaatctt	78
		R	ccacaagccttactcgtgaga	
*Oct4*	NM_001252452.1	F	cacgagtggaaagcaactca	82
		R	gctttcatgtcctgggactc	
*Zar1*	NM_174877.3	F	ctcaggaccccggtgatt	15
		R	cactcggcagaactgtttga	
*Luciferase*	M15077.1	F	gtcttcccgacgatgacg	70
		R	gtctttccgtgctccaaaac	

### Extension Poly(A) Test (ePAT)

To measure poly(A) tail length in aged oocytes compared to controls, ePAT was performed according to Jänicke et al. [37]. A PAT-anchor primer consisting of a unique universal DNA segment and a 22-T stretch was annealed to the 3′ end of adenylated RNA and served as a DNA-template for the Klenow-fragment. This polymerase introduces the PAT-anchor primer to the end of the poly(A) tail following standard reverse transcription from this anchor, and therefore includes the poly(A) tail of the RNA. Subsequently amplification with a primer complementary to the universal DNA segment and a gene specific primer was performed. This resulted in PCR-fragments with variable sizes based on the absolute length of the poly(A) tail of the mRNA. In the current study, two representative ME genes (*Zar1* and *Dnmt1*) were used to confirm possible poly(A) tail changes in aged oocytes compared to controls, as indicated by qRT-PCR. Total RNA of 200 postovulatory-aged and of 200 control oocytes matured in vivo was denatured in the presence of 1 µl of 100 mM PAT-anchor primer (5′-GCGAGCTCCGCGGCCGCGTTTTTTTTTTTT-3′) at 80°C for 5 min. Then RT-master mix (4 µl 5× Superscript III Buffer, 1 µl 100 mM DTT, 1 µl 10 mM dNTPs, 1 µl RNaseOUT, 5 U Klenow-fragment) was added and the samples incubated at 37°C for 1 h followed by inactivation at 80°C for 10 min and cooling down to 55°C for 1 min. Subsequently, reverse transcription was performed by adding 1 µl Superscript III (Life Technologies), incubation at 55°C for 1 h and inactivation at 80°C for 10 min. PCR was conducted using ReadyMix Taq PCR Reaction Mix (Sigma-Aldrich) under the following conditions: initial denaturation at 93°C for 5 min, amplification by 35 cycles of 30 s at 93°C, 60 s at 60°C, 60 s at 72°C, and 72°C for 10 min. The PAT-anchor primer and the following gene-specific primers (Eurofins) were used: *Zar1* 5′-CTAGATGGGGCTAATGGAATGG-3′; *Dnmt1* 5′-CACTGTGCAGGTGGCAAG-3′. Extension PAT-fragments with variable poly(A) tail lengths were detected on a 2% agarose gel. In addition, PCR products were separated by capillary electrophoresis and fluorescence intensity measured using the Agilent 2100 Bioanalyzer with a DNA 1000 chip (Agilent Technologies).

### Identification of potential CPEs

The 3'UTR sequence of the ten ME genes investigated was obtained from the Ensemble databank (www.ensemble.org) via Ref-Seq transcript ID. By using an exact pattern-matching approach, implemented in the Perl programming language, cytoplasmic polyadenylation element (CPE) motif position and polyadenylation signal (PAS) motif (aauaaa) position were computed. The algorithms searched for the following seven CPE sequences: uuuaau, uuuuau, uuuuauu, uuuuaaau, uuuuuaau, uuuuuau and uuuuuuau. CPE motifs were only taken into account when located within 150 bp of the PAS. When the sequence contained more than one PAS, the motif located closest to the 3′ end of the transcript was chosen for further analysis.

### Statistical analysis

Quantitative RT-PCR results were analyzed using SigmaPlot version 12.5 (Systat Software, Inc). A two-tailed Student *t*-test was performed to analyze the statistical significance of differences between aged and control oocytes and comparisons of fold change (2^-ΔΔCt^) of oligo(dT)_16_-primed cDNA with random hexamer-primed cDNA. Jarque-Bera and Kruskal-Wallis tests were employed for stage-specific follicle growth, Chi^2^-test for culture survival and maturation rate and the Mann-Whitney U-test were used for analysis of estrogen and progesterone concentrations. The α-level was set at 0.05 to determine statistically significant differences.

## Results

### Pre- and postovulatory aging in in vitro-grown oocytes

**Figure 2:**
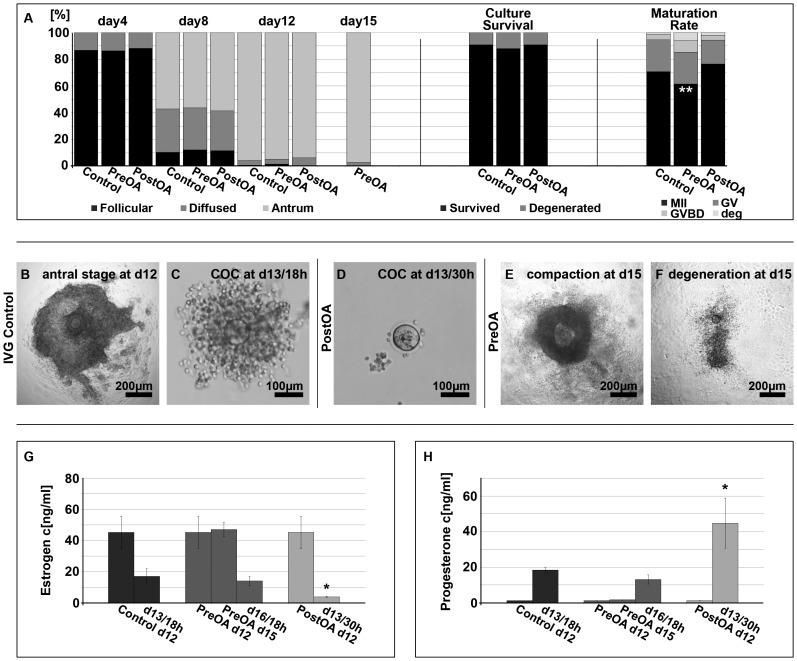
Follicle morphology, morphokinetics, and hormone concentrations in conditioned medium of preantral follicle culture. A) Follicle characteristics, culture survival and maturation of control (*n* = 1230), preovulatory-aged (PreOA; *n* = 411) and postovulatory-aged (PostOA; *n* = 613) oocytes. B-D) Antral stage follicle grown in vitro for 12 d (B) and cumulus-oocyte complexes on day 13 after in vitro ovulation in control oocytes (C) and after postovulatory aging (D) for 12 h. E, F) Altered granulosa cell characteristics after preovulatory aging at day 15 of culture; follicles with an increased accumulation of mural granulosa cells and an apparent follicle compaction (E), and a degenerating follicle with dispersed granulosa cells and a nearly denuded oocyte from day 15 of culture (F). G) Estrogen and (H) progesterone levels (mean ± SEM) in conditioned culture medium prior to and past hormonal stimulation by rhCG/rEGF in the different experimental groups (* *P*<0.05, ** *P*<0.01).

Follicle development from preantral to antral stage before priming for in vitro ovulation was homogenous among all groups (Fig. 2A; left panel). However, follicle degeneration was slightly increased after preovulatory aging for three days compared to controls (Fig. 2A; middle panel) although rates did not reach statistical significance. In controls, 70.7% of follicles possessed oocytes with first polar body at the end of culture on day 13 (*n = *691/977; Fig. 2A; right panel). This rate increased to 76.5% after postovulatory aging for 12 h (*n = *342/447). In contrast to postovulatory aging, the rate of in vitro ovulated oocytes that emitted a first polar body was significantly reduced to 61.4% (*P<*0.01) after preovulatory aging for three days (*n = *198/322) compared to controls.

Analysis of follicle morphology showed that follicles developed regularly to the antral stage at day 12 (Fig. 2B). However, cumulus cells frequently detached from oocytes after in vitro ovulation on day 13 followed by postovulatory aging for 12 h compared to controls (30 h instead of 18 h post rEGF/hCG; Fig. 2C and 2D). Additionally, preovulatory aged follicles exhibited altered granulosa cell characteristics at day 15 compared to day 12. This was indicated by an increased accumulation of mural granulosa cells and an apparent follicle compaction (Fig. 2E) or in some cases by the dispersal of granulosa cells of the follicle and partial or total loss of cumulus cell attachment, oocyte expulsion and follicle degeneration (Fig. 2F).

Hormone levels in conditioned culture medium did not differ between groups up to day 12, indicative of robust hormone output. However, E2 concentration in supernatant became significantly decreased after postovulatory aging for 12 h (3.7 ng/ml; *P<*0.05) compared to controls on day 13 (16.9 ng/ml; Fig. 2G). Progesterone levels were low at day 12 prior to stimulation by hCG/rEGF in all groups (<2 ng/ml), and stayed low after preovulatory aging in vitro for three days. As expected, progesterone concentration became increased in all groups after hormonal stimulation by rhCG/rEGF. Progesterone increased significantly during postovulatory aging for 12 h (44.7 ng/ml; *P = *0.05), and was slightly but not significantly lower after in vitro ovulation following preovulatory aging for three days (13.1 ng/ml) compared to controls (18.3 ng/ml; Fig. 2H).

Thus, preovulatory aging in vitro leads to slightly increased follicle degeneration, altered granulosa cell characteristics and reduced nuclear maturation competence. Analysis of steroid production indicates an increased progesterone production and over-luteinization after postovulatory aging in follicle culture.

### Increase of poly(A) tail length of mRNA in preovulatory-aged oocytes

**Figure 3:**
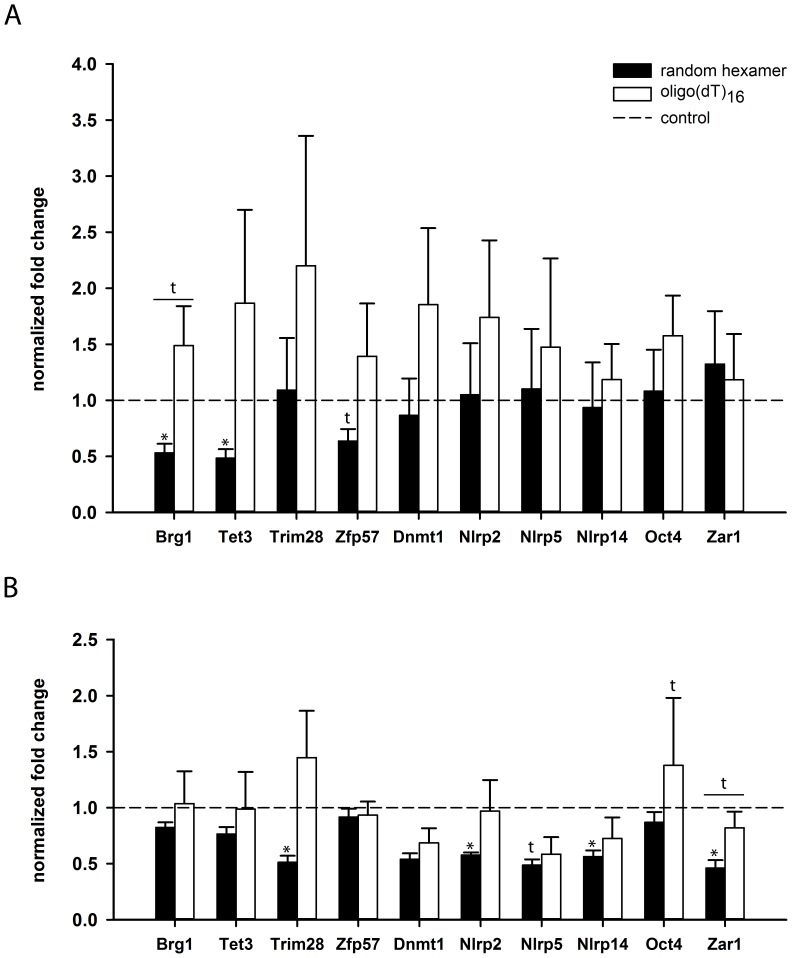
Expression levels and poly(A) content of ME genes in preovulatory-aged oocytes. The normalized fold change (mean ± SEM) of preovulatory-aged oocytes compared to control oocytes (dotted line) of total transcript (black bars) and polyadenylated transcript (white bars) is shown. A) After preovulatory in vivo aging, oocytes show a significant decline in total transcript levels for *Brg1* and *Tet3*. Comparison of total with polyadenylated transcript levels reveals that poly(A) content of ME gene mRNA tends to increase during preovulatory in vivo aging. B) Total transcript amounts of *Trim28*, *Nlrp2*, *Nlrp14* and *Zar1* decreased significantly after preovulatory aging in vitro. A similar trend was observed for *Nlrp5*. Several genes investigated showed a tendency towards a relative increase in poly(A) content compared to total transcript levels, which was most evident for *Zar1* (t: *P*<0.10, * *P*<0.05).

Poly(A) tail dynamics of ME gene mRNAs were analyzed in oocytes after preovulatory aging in vivo as well as in vitro (Fig. 3). The total amount of transcripts was determined by qRT-PCR analysis using random-hexamer primers. Changes in poly(A) tail length were evaluated by comparing expression fold changes of random hexamer-primed cDNA with those of oligo(dT)_16_ primed-cDNA. After induction of in vivo preovulatory aging by delaying ovulation for three days, *Brg1* and *Tet3* showed significantly lower total transcript levels in MII oocytes compared to controls (*P<*0.05) and a trend towards declining transcript levels for *Zfp57* (*P* = 0.0620). No significant changes in transcript amount were observed for *Trim28*, *Dnmt1*, *Nlrp2*, *Nlrp5*, *Nlrp14*, *Oct4* and *Zar1* (Fig. 3A, black bars). In vivo preovulatory aging had no significant effect on poly(A) tail length of ME gene mRNAs. However, there was a tendency towards a relative increased poly(A) tail length for all genes investigated, except for *Zar1*, as indicated by an increase of oligo(dT)_16_-primed cDNA compared to random hexamer-primed cDNA (Fig. 3A, white bars).

Delaying ovulation for three days in vitro caused a significant decline in total mRNA content of MII oocytes for *Trim28*, *Nlrp2*, *Nlrp14* and *Zar1* compared to controls (Fig. 3B, black bars). Similar to preovulatory aging in vivo, preovulatory aging of oocytes in vitro caused a tendency towards a relative increase in poly(A) tail length for all genes investigated. A trend for this polyadenylation was observed for *Zar1* (*P = *0.0752; Fig. 3B).

### Decrease of poly(A) tail length of mRNA in postovulatory-aged oocytes

**Figure 4:**
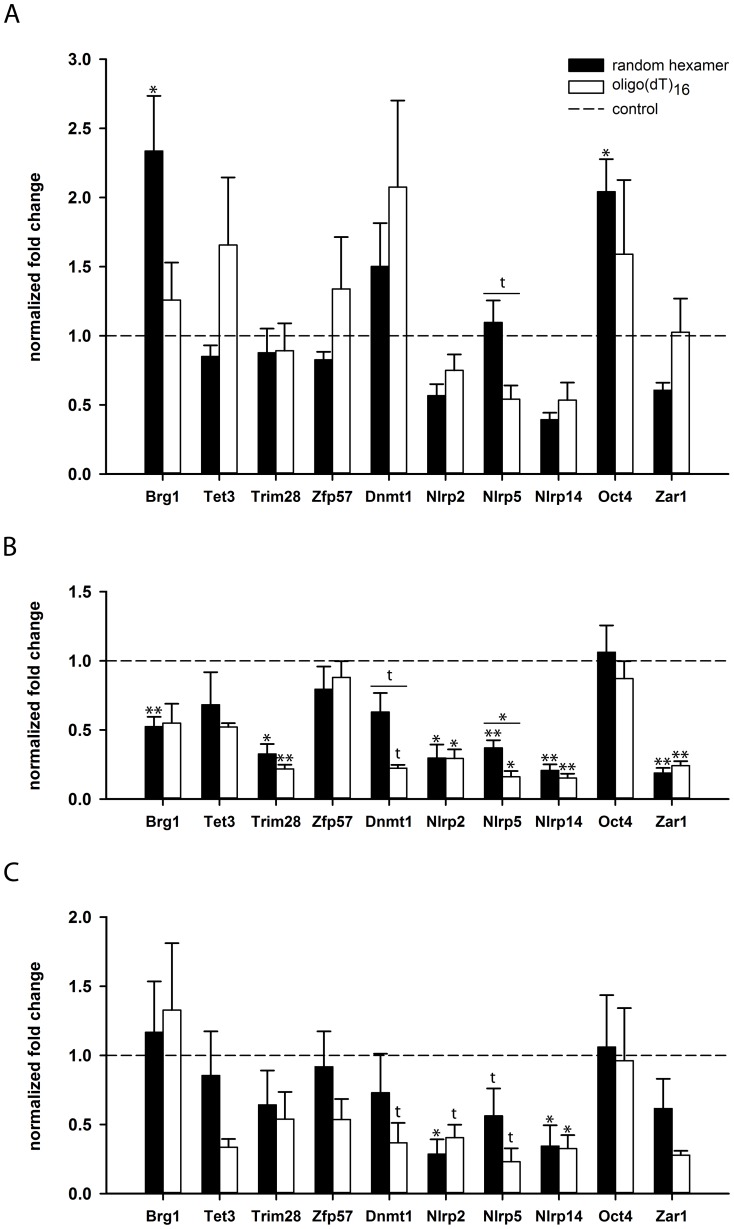
Expression levels and poly(A) content of ME genes in postovulatory-aged oocytes. The normalized fold change (mean ± SEM) of postovulatory-aged oocytes compared to control oocytes (dotted line) of total transcript (black bars) and polyadenylated transcript (white bars) is shown. In vivo-grown oocytes were aged for 12 h (A) and 24 h (B). After 12 h of postovulatory aging, the stronger reduction of polyadenylated transcript in comparison to total transcript levels indicates a reduced poly(A) tail length for *Nlrp5*. After 24 h, postovulatory aging results in a considerable decline of overall transcript amount and a decline in poly(A) content for most of the genes investigated. This decline in poly(A) content was significant for *Nlpr5* and showed a trend for *Dnmt1*. C) Poly(A) content of in vitro-grown oocytes tended to decline for eight of the ten genes investigated already after 12 h of postovulatory aging, (t: *P*<0.10, * *P*<0.05, ** *P*<0.01).

We analyzed the effect of 12 and 24 h in vivo postovulatory aging on poly(A) tail dynamics of selected ME genes (Fig. 4). After 12 h of postovulatory aging, a decline in oligo(dT)_16_-primed cDNA compared to random hexamer priming was seen for *Nlrp5* (*P = *0.0577), indicating a poly(A) tail reduction of the transcripts of this gene (Fig. 4A). For all other genes investigated, no significant effects of postovulatory aging on poly(A) tail length were observed. However, *Brg1* and *Oct4* showed an increase in total transcript levels after aging in comparison to controls (Fig. 4A), for which we do not have an explanation yet as oocytes should be transcriptionally inactive [2].

More distinct effects were observed in in vivo-grown oocytes after 24 h of postovulatory aging. This prolonged aging resulted in significantly decreased levels of both random hexamer-primed and oligo(dT)_16_-primed cDNA for *Trim28, Nlrp2*, *Nlrp5*, *Nlrp14* and *Zar1* and of random hexamer-primed cDNA for *Brg1* compared to controls (*P<*0.05). Comparison of random hexamer and oligo(dT)_16_ priming demonstrated a significant poly(A) tail shortening during 24 h postovulatory aging for *Nlrp5* (*P<*0.05) and a trend for a decreased poly(A) tail length for *Dnmt1* (*P = *0.0518; Fig. 4B).

Gene expression and poly(A) tail dynamics of ME genes were also analyzed in 12 h postovulatory-aged oocytes from in vitro-grown oocytes that aged within the follicle surrounded by cumulus cells before oocyte retrieval (Fig. 4C). For several genes investigated, a decline of total transcript level was observed after postovulatory aging compared to controls. This was significant for *Nlrp2* and *Nlrp14* (*P<*0.05). Moreover, we saw significantly lower amounts of oligo(dT)_16_-primed cDNA for *Nlrp14* (*P<*0.05) after postovulatory aging compared to controls, a trend for lower amounts of *Dnmt1* (*P = *0.0864), *Nlrp2 (P = *0.0736) and *Nlrp5 (P = *0.0896), and a tendency for *Tet3*, *Trim28*, *Zfp57* and *Zar1*. In addition, a tendency in reduction of oligo(dT)_16_-primed cDNA compared to random hexamer-primed cDNA was observed for *Tet3*, *Trim28*, *Zfp57*, *Dnmt1*, *Nlrp5* and *Zar1*, indicating shortening of poly(A) tail length of the mRNA of these genes (Fig. 4C).

### Confirmation of poly(A) tail changes of RNA-transcripts in aged oocytes

**Figure 5:**
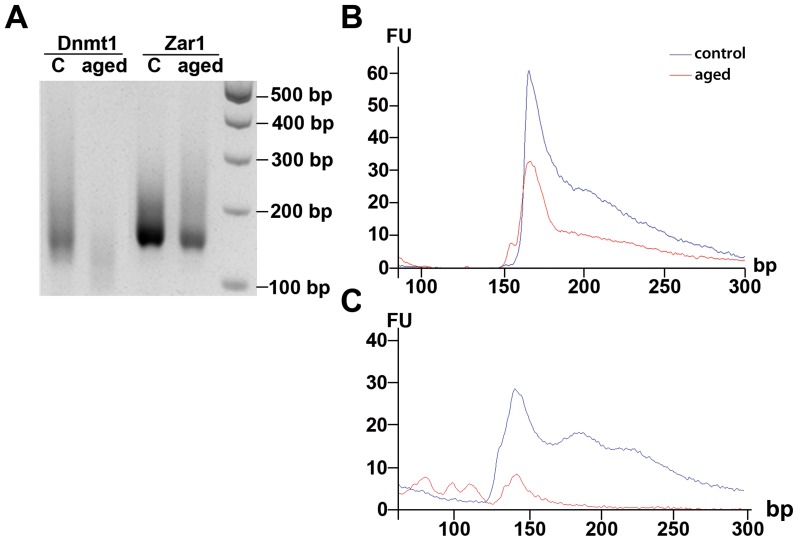
Quantification of poly(A) tail length for *Dnmt1* and *Zar1* by ePAT. 24 h postovulatory-aged, in vivo-grown oocytes were analyzed by extension poly(A) test (ePAT). A) Gel electrophoresis of the product shows a decrease of poly(A) tail length for *Dnmt1* in aged oocytes compared to controls, whereas poly(A) tail length of *Zar1* remains widely stable. These results were quantified by capillary electrophoresis for *Zar1* (B) and *Dnmt1* (C). Indicated is the fluorescence intensity (FU) of amplicon lengths (in base pairs) for aged oocytes (red line) and controls (blue line).

To validate the poly(A) tail changes indicated by the qRT-PCR analysis, we performed extension poly(A) test (ePAT) according to the method described by Jänicke et al. [37]. This method allows to measure the length of the poly(A) tail of mRNA of a specific gene. Since PCR fragments with variable sizes depending on the length of the poly(A) tail of the mRNA are produced, the outcome results in a smear on an agarose gel which represents the differences in poly(A) tail length. To verify the results of the qRT-PCR analysis by determining the exact poly(A) tail length, two representative genes of in vivo-grown MII oocytes were analyzed by ePAT after 24 h of postovulatory aging: *Zar1*, which showed no significant changes in poly(A) tail length in qRT-PCR, and *Dnmt1*, for which a poly(A) tail reduction was indicated. *Dnmt1* showed a major degradation of total mRNA after postovulatory aging represented by decreased smear intensity in the aged group compared to controls (Fig. 5A). In addition, mRNA of *Dnmt1* revealed a poly(A) tail shortening in postovulatory-aged oocytes compared to controls indicated by a shift of the signal for PCR products to smaller fragment sizes. For *Zar1*, ePAT analysis again revealed mRNA degradation, but no obvious poly(A) tail changes in 24 h postovulatory-aged oocytes (Fig. 5A).

In addition to agarose gel electrophoresis, products resulting from the ePAT-assay were analyzed by capillary electrophoresis on an Agilent 2100 Bioanalyzer with a DNA 1000 chip, allowing a more precise quantification of the poly(A) tail length than gel electrophoresis. For *Zar1*, a reduction in total amount of transcript was observed in postovulatory-aged oocytes as indicated by declining from 5.33 ng/µl in controls to 2 ng/µl in aged oocytes (Fig. 5B). The amplicon length for *Zar1* without a poly(A) tail is 165 bp. For both experimental groups, the maximum peak of the *Zar1* PCR product was detected at about 170 bp, with a continuous increase in PCR products up to a size of about 300 bp, pointing to a poly(A) tail of 5 to 130 nucleotides. Thus, *Zar1* revealed hardly any poly(A) tail reduction after postovulatory aging of oocytes. For *Dnmt1*, a stronger decrease in total mRNA was observed as indicated by declining in mRNA concentration from 5.17 ng/µl in controls to amounts too low to be quantified (Fig. 5C). The amplicon length for *Dnmt1* without poly(A) tail is 100 bp. In control oocytes, the main fraction of the PCR product was detected at about 140 bp with a continuous increase in PCR products up to a size of about 300 bp, pointing to a poly(A) tail of 40 to 200 nucleotides. After 24 h of postovulatory aging, a clear shift towards shorter fragments down to 100 bp was observed for *Dnmt1* (Fig. 5C), confirming the poly(A) tail reduction demonstrated by qRT-PCR.

### Identification of potential CPEs

Only three out of the ten ME genes investigated (*Brg1*, *Tet3* and *Dnmt1*) contain at least one consensus CPE motif within 150 bp of the polyadenylation signal (PAS) motif (Table 2). In all three genes, more than one copy of the PAS motif was found by the algorithm, but only the closest to the 3′ end of the transcript was used for the CPE analysis. *Brg1* and *Dnmt1* 3′UTRs encompass one CPE sequence each. *Tet3* contains three different CPE sequences within 150 bp of the PAS that is closest to the 3′ end.

**Table 2:** Sequence and position of CPEs of ME-genes within 150 bp of the PAS.

Gene	Ref-Seq transcript ID	Position PAS (aauaaa)	CPE sequence	CPE position
*Brg1*	NM_011417.3	6340*	uuuuaaau	6323
*Tet3*	NM_183138.2	10876*	uuuuaau	10872
			uuuuau(u)	10798
			uuuuau	10882
*Trim28*	NM_011588.3	3222	-	-
*Zfp57*	NM_001013745.2	1570	-	-
*Dnmt1*	NM_001199431.1	5346*	uuuuau	5320
*Nlrp2*	NM_117690.3	3382*	-	-
*Nlrp5*	NM_001039143.1	3489*	-	-
*Nlrp14*	NM_001002894.2	1424*	-	-
*Oct4*	NM_001252452.1	1324	-	-
*Zar1*	NM_174877.3	1239	-	-

*Position of PAS-sequence lying closest to 3′ end of the transcript.

## Discussion

We analyzed the effect of pre- and postovulatory aging on transcript levels and poly(A) tail length of ten ME genes in murine MII oocytes. In vivo preovulatory aging was investigated in an established mouse model [14]. For in vitro preovulatory aging, we validated a previously established murine follicle culture model [31], [35] and showed that we could successfully delay the time point of ovulation in this in vitro model. Oocyte-secreted factors prevent premature luteinization and progesterone increase in the follicle [33]. This also occurred during preovulatory aging in follicle culture, as progesterone remained low prior to stimulation with rhCG/rEGF, but there was a dramatically increased progesterone level after postovulatory aging. Analysis of hormone production and follicle morphology indicate that pre- and postovulatory aging influence follicular homeostasis, steroidogenesis and gene expression.

In vivo preovulatory aging of oocytes induced by delayed ovulation resulted in reduced transcript levels of *Brg1*, *Tet3* and *Zfp57*. *Brg1* encodes the catalytic subunit of SWI/SNF-related complexes involved in chromatin remodeling during ZGA, and thus, is necessary for the progression of the embryo beyond the 2-cell stage [8]. *Tet3* catalyzes the oxidation of 5-methylcytosine (5mC) to 5-hydroxymethylcytosine (5hmC) and has been shown to regulate processing of the paternal genome [7]. *Zfp57* is responsible for post-fertilization protection and maintenance of DNA methylation of imprinted genes [38]. Surprisingly, we observed a trend towards increased poly(A) tail lengths for most of the genes investigated in in vivo preovulatory aged oocytes. The cytoplasmic regulation of mRNA adenylation is an important mechanism of posttranscriptional control in oocytes, since transcription is repressed from prophase I of the first meiotic cell division up to ZGA [39], [40], [41]. The best understood mechanism of translational recruitment by cytoplasmic polyadenylation is mediated by a CPE lying close to the nuclear PAS (aauaaa) in the 3′-UTR [5], [41]. Among the transcripts demonstrating an increase in poly(A) tail length after in vivo preovulatory aging only *Brg1*, *Tet3* and *Dnmt1* have a CPE, suggesting CPE-dependent polyadenylation of these transcripts. However, this cannot be the only polyadenylation mechanism, because *Zfp57* also showed polyadenylation, but does not contain a CPE sequence [42]. Maximal polyadenylation of 250–300 poly(A) residues occurs within the nucleus, and shortening usually takes place after transport to the cytoplasm [27]. Prolonged meiotic arrest may cause increased readenylation of mRNAs in the cytoplasm during preovulatory aging and maturation to MII. Although we observed high standard deviations for qRT-PCR products of oocyte mRNA resulting in significant changes only for some of the genes, a concordant effect on the adenylation status was seen for almost all of the genes investigated. Our data point to increased polyadenylation of transcripts during in vivo preovulatory aging of oocytes, which might result in precocious recruitment of ME proteins. Increased poly(A) tail length has been described before for *BUB1B* and *MAD2L1* mRNAs, coding for spindle and cell cycle regulating factors, in in vitro-matured human oocytes that have low developmental competence [27]. It remains to be evaluated whether increased polyadenylation contributes to an altered proteome, and thus, a reduced oocyte competence as indicated by an increase in embryonic resorption sites after delayed ovulation in this mouse model [14].

After in vitro preovulatory aging, total transcript levels of *Trim28*, *Nlrp2*, *Nlrp14* and *Zar1* declined significantly in MII oocytes, and an accordant trend was observed for *Nlrp5*. *Trim28* encodes a scaffolding protein that interacts with ZFP57 and has a central role in genomic imprint maintenance during preimplantation embryonic development [9]. The exact function of *Nlrp2* and *Nlrp14* is currently unknown, but knock-outs in oocytes lead to embryonic arrest at the 2-cell stage [43], [44]. It is tempting to speculate that a deficiency of *Nlrp2* and *Nlrp14* may also lead to imprinting defects, which are observed as a consequence of *NLRP2* and *NLRP7* mutations in humans [45], [46].

A recent study demonstrated no significant changes in the total mRNA amount for the ME genes *Dnmt3a*, *Dnmt3l* and *Zfp57* in preovulatory-aged IVM oocytes in the mouse [47]. In line with this, we also did not observe a change in *Zfp57* expression after in vitro preovulatory aging. Our study shows, however, that the consequences of in vivo preovulatory aging are different from those of in vitro preovulatory aging. Such a diverse regulation of transcript levels has been shown before by Sánchez and coworkers, who described a significant decrease in total mRNA amount of *Bmp-15*, *Gdf-9*, *Nlrp5*, *Zar1* and *Fgf-8* in immature in vitro-grown oocytes compared to in vivo-grown oocytes [48]. In our study, *Zar1*, an oocyte-specific transcription factor [49], shows not only a significant loss of total mRNA content in in vitro preovulatory aged oocytes compared to controls, but also a distinct trend towards poly(A) tail elongation. In general, poly(A) tail length tended to increase after in vitro preovulatory aging, which was in line with results from oocytes after in vivo preovulatory aging. Nevertheless, differences in amount of transcripts were observed when comparing in vivo and in vitro preovulatory aged oocytes. This suggests an effect of environmental factors that influence mRNA stability and poly(A) tail dynamics in the oocyte, which are different in the ovary and in follicle culture. These and previous findings may explain why oocytes from follicle culture are still of lower quality than in vivo-grown oocytes [50], in spite of improved culture methods.

It has long been known that successful fertilization in the mouse has to occur within the first 15 h after ovulation [51], and that extended postovulatory aging leads to decreased implantation rates, lower litter sizes and pups with retarded growth [20]. Likewise, delayed ovulation in humans is discussed to be involved in delayed implantation, implantation failures and suboptimal uterine receptivity [52], [53]. We observed a significant total transcript level reduction of most of the transcripts studied after 24 h of postovulatory aging of in vivo-matured MII mouse oocytes. In addition, we observed a reduction of the poly(A) tail length of *Dnmt1* and *Nlrp5* transcripts; for *Nlrp5* this trend was already seen after 12 h. Such a poly(A) tail shortening could lead to reduced rates of translation and/or mRNA degradation [3], [54], [55]. Thélie and colleagues found a significant decline in poly(A) tail length for *Nlrp5* and *Zar1* during in vivo and in vitro maturation of bovine oocytes, but not during preimplantation events [36]. We could confirm a decline in *Nlrp5* poly(A) tail length, but not *Zar1* poly(A) tail length, after postovulatory aging. Postovulatory aging of in vitro-grown oocytes in follicle culture resulted in a major mRNA decrease of most of the ME genes investigated already after 12 h, whereas the effects of in vivo aging were only significant after 24 h. Thus, oocytes in follicle culture may be more susceptible to age-related deadenylation of ME genes than oocytes grown in vivo. Also, presence of cumulus cells and culture environment may affect the postovulatory aging processes [56], [57], [58]. Like after postovulatory aging in vivo, *Dnmt1* and *Nlrp5* transcripts appeared to be more susceptible to deadenylation compared to other transcripts in vitro. *Nlrp5* is an essential part of the subcortical maternal complex that is located at the plasma membrane of the oocyte, and it is involved in mitochondrial activation, endoplasmic reticulum localization and calcium homeostasis in oocytes and early embryos [59], [60], [61], [62]. Poly(A) tail shortening of *Nlrp5* mRNA could lead to impaired protein translation and subsequently disturb fertilization and preimplantation development of the embryo. *Dnmt1*, like *Trim28* and *Zfp57*, is known to be involved in protection of genomic imprints and their maintenance during preimplantation development [9], [38], [63], [64]. The observed reduction of poly(A) tail length of *Dnmt1* during postovulatory aging may reduce its translation, and thereby, impair imprint maintenance during preimplantation development. Thus, deadenylation of ME gene transcripts during postovulatory aging may contribute to the decreased developmental competence of aged oocytes.

We have recently shown that postovulatory aging of eggs from *Xenopus tropicalis* led to deadenylation of transcripts involved in translation and energy metabolism, decreased fertilization rates and increased malformation rates of the larvae [28]. Here, we describe for the first time changes in poly(A) tail length after aging of mammalian oocytes. In addition, our study demonstrates that there is a profound difference in poly(A) tail dynamics between pre- and postovulatory aging of oocytes. While polyadenylation of ME gene transcripts was observed during preovulatory aging, postovulatory aging caused their deadenylation. Our findings suggest that RNA and poly(A) tail turn-over are not or not only triggered by ovulation or fertilization, but timed, at least to some degree, by events in the growing, still meiotically arrested oocyte or in the maturing oocyte. Thus, delayed ovulation and fertilization may desynchronize the oocyte and in consequence the embryonic programs with potentially long-lasting effects like reduction in reproductive fitness and longevity [65].

Since in vivo pre- and postovulatory aging can occur in the course of assisted reproduction, and cryopreservation of follicles followed by in vitro culture is being considered as an option for fertility preservation, these results may be of clinical relevance.
